# Effects of tumor necrosis factor-α polymorphism on the brain structural changes of the patients with major depressive disorder

**DOI:** 10.1038/s41398-018-0256-x

**Published:** 2018-10-11

**Authors:** Rubai Zhou, Fan Wang, Guoqing Zhao, Weiping Xia, Daihui Peng, Ruizhi Mao, Jingjing Xu, Zuowei Wang, Wu Hong, Chen Zhang, Yong Wang, Yousong Su, Jia Huang, Tao Yang, Jijun Wang, Jun Chen, Lena Palaniyappan, Yiru Fang

**Affiliations:** 1 Division of Mood Disorders, Shanghai Mental Health Center, Shanghai Jiao Tong University School of Medicine, Shanghai, China; 2 CAS Center for Excellence in Brain Science and Intelligence Technology, Shanghai, China; 3 Shanghai Key Laboratory of Psychotic disorders, Shanghai, China; 4 Department of EEG & Neuroimaging, Shanghai Mental Health Center, Shanghai Jiao Tong University School of Medicine, Shanghai, China; 5 Bio-X Institutes, Key Laboratory for the Genetics of Developmental and Neuropsychiatric Disorders (Ministry of Education), Shanghai, China; 6 Brain Science and Technology Research Center, Shanghai Jiao Tong University, Shanghai, China; 7 Robarts Research Institute& The Brain and Mind Institute, University of Western Ontario, London, ON Canada; 8 Department of Psychiatry, University of Western Ontario, London, ON Canada; 9 Lawson Health Research Institute, London, ON Canada; 10 Hongkou District Mental Health Center of Shanghai, Shanghai, China; 11 Department of Psychology, Provincial Hospital Affiliated to Shandong University, Jinan, 250021 China; 12 Department of Medical Psychology, Xinhua Hospital, Shanghai Jiao Tong University School of Medicine, Shanghai, China

## Abstract

Single Nucleotide Polymorphic (SNP) variations of proinflammatory cytokines such as Tumor Necrosis Factor-α (TNF-α) have been reported to be closely associated with the major depressive disorder (MDD). However, it is unclear if proinflammatory genetic burden adversely affects the regional gray matter volume in patients with MDD. The aim of this study was to test whether rs1799724, an SNP of TNF-α, contributes to the neuroanatomical changes in MDD. In this cross-sectional study, a total of 144 MDD patients and 111 healthy controls (HC) well matched for age, sex and education were recruited from Shanghai Mental Health Center. Voxel-based morphometry (VBM) followed by graph theory based structural covariance analysis was applied to locate diagnosis x genotype interactions. Irrespective of diagnosis, individuals with the high-risk genotype (T-carriers) had reduced volume in left angular gyrus (main effect of genotype). Diagnosis x genotype interaction was exclusively localized to the visual cortex (right superior occipital gyrus). The same region also showed reduced volume in patients with MDD than HC (main effect of diagnosis), with this effect being most pronounced in patients carrying the high-risk genotype. However, neither global nor regional network of structural covariance was found to have group difference. In conclusion, a genetic variation which can increase TNF-α expression selectively affects the anatomy of the visual cortex among the depressed subjects, with no effect on the topographical organization of multiple cortical regions. This supports the notion that anatomical changes in depression are in part influenced by the genetic determinants of inflammatory activity.

## Introduction

Major depressive disorder (MDD) is associated with a high rate of morbidity, recurrence, disability and mortality^1,2^. Despite being a leading cause of global disease burden^3^, pharmacological strategies of MDD so far have been restricted to the manipulation of monoamine neurotransmission, with only partial effect on the symptom burden.

A growing evidence has suggested that peripheral and central inflammatory response may play an important role in the pathogenesis and development of MDD, especially cytokines^4^. Tumor necrosis factor-α (TNF-α) is a pro-inflammatory cytokine produced largely by macrophages^5^, and is involved in both neurotoxic effects as well as neuroprotective effects^6^. Overexpression of TNF-α is deleterious in the early stage of tissue damage, but it can aid recovery later^7^. Pathophysiologically, elevated TNF-α may arouse lessened neuronal synaptic plasticity, reduced neurotropic factors and declined neurogenesis^8,9^. It has been reported that patients with MDD have enhanced plasma levels TNF-α, and antidepressant treatments can decrease this levels^10,11^. Moreover, increasing evidence has shown anti-TNF-α therapy could help to relieve depressive symptoms and repair cognitive impairments^12^. Yang et al.^13^. have shown peripheral TNF-α can affect the brain structure via dendritic elimination independent of central inflammatory activity by using a preclinical mouse model. In particular, inhibition of peripheral TNF-α action prevents structural changes at the synaptic level. The peripherally produced cytokines are also able to act on the brain through deficient blood-brain-barrier, active transport via saturable transport molecules, activating endothelial cells to generate second messengers, and binding to receptors on afferent nerve fibers^4,14–16^.

The rs1799724 (-850 or 857 C/T) is a functional single nucleotide polymorphism (SNP) located in the promoter regions of TNF-α gene (GenBank accession no. NG_007462, transcript id NM_000594.3), which can affect the genetic transcriptional activity of TNF^17^. The high-risk genotype (-857C/T) of rs1799724 has been found to give rise to higher levels of mRNA and protein than the low-risk genotype (−857C/C)^17^. It has been proved that rs1799724 is an important SNP closely associated with neurological disorders like Alzheimer’s disease^18,19^, stroke^20^ and depression after stroke^21^. In the presence of post-stroke depression, serum TNF-α concentration is higher in T-carriers (high-risk genotype) than in non-T carriers (low-risk genotype), indicating that 805 C/T genotype moderates serum TNF-a levels especially in the presence of depression^21^.

In recent times, several lines of evidence have implicated cytokines to have neurotrophic functions that help to both promote and prune neuronal synapses, thus serving as experience-dependent plasticity regulators both during development and in adult life^22,23^. In particular, TNF-α has been found critical to the development of unimodal sensory cortex, especially visual^24^, auditory^25^ and somatosensory^13^ cortices. It has been proved by neuroimaging studies that peripheral TNF-α levels are negatively linked to regional volumes in the left occipitotemporal area, left superior occipital gyrus, left inferior parietal lobule and bilateral medial prefrontal cortices of healthy people^26^. Some other SNPs of TNF-α and its receptors have been revealed to affect brain volume of hippocampus, striatum and caudate in healthy people^27,28^.

In this context, we aimed to evaluate the effects of functional TNF-α SNP, rs1799724, on brain structure in both MDD patients and healthy controls using voxel-based morphometry (VBM). We also sought diagnosis x genotype interaction and followed this up with graph analysis to determine the systemic impact of any localized changes.

## Materials and methods

### Participants

According to a previous study^29^, the minimum sample size in each group should be 80 to guarantee 80% voxel-based power for grey matter analysis, and 144 patients with MDD and 111 healthy controls were included in this study finally. The patients, who met Diagnostic and Statistical Manual of Mental Disorders (DSM)–IV-TR criteria for MDD, were recruited from the clinics of Shanghai Mental Health Center (SMHC). They were 20–40 years old, right-handed, and educated up to or above junior high school level. All patients were medication-free when they were scanned, including 80 drug-naïve first-episode patients, 27 drug-naïve multi-episode patients, and 37 multi-episode patients who were not medicated for at least one month before the scanning. Moreover, when assessed by Hamilton’s Depression Scale with 17 items, the patients included should be with total scores no less than 17 and scores of depressive mood item no less than 2. Those patients with other axes I disorders and debilitating general medical disorders were excluded. Age, sex, and education matched HCs, who could pass our screening and had no history of psychosis or debilitating general medical disorders, were also recruited. This study was approved by the Institutional Review Board of Shanghai Mental Health.

Centre after strict inspection of the protocol, and all the participants have written informed consents. (Clinical Trial Registry Number: NCT01764867, http://www.clinicaltrials.gov)

### Genotyping

The genomic DNA was isolated from the peripheral blood using Lifefeng Blood DNA Kit (Shanghai Lifefeng Biotech Co., Ltd, China). The SNP rs1799724 of TNF-α was genotyped via the Multiplex SnaPshot technique (Genesky Biotechnologies, Inc., Shanghai, China). The PCR primer was rs9964_rs9724F/R. All the 255 subjects were successfully genotyped for this promoter polymorphism of TNF-α. Hardy–Weinberg equilibrium analysis for the single SNP was performed using Chi-squared Test with SPSS19.0 (SPSS Inc., Chicago, IL, USA). The SNP rs1799724 genotype distribution of all the subjects was in Hardy–Weinberg equilibrium (*p* = 0.806).

### Magnetic resonance imaging (MRI)

All the MRI data were obtained in the radiology department of SMHC using a 3.0 T Siemens scanner and high-resolution T1 images were used in the analyses of the present study. Three-dimensional magnetization prepared rapid acquisition gradient echo sequence (3D-MPRAGE) had repetition time = 2530 ms, echo time = 3.65 ms, flip angle α = 7°, 224 sagittal slices, field of view = 256 × 256 mm, matrix = 256 × 256, thickness = 1 mm. Images were checked out for movement artifacts and homogeneity of image intensity immediately after scans and repeated if necessary. We also collected a clinical diagnostic MR sequence in the same session and excluded individuals who had ischemia, cavum septum pellucidum, radiologically notable cerebral leukomalacia as determined by a radiologist.

### Voxel-based morphometry (VBM)

We used VBM8^30^ (Department of Psychiatry, University of Jena) toolbox based on Statistical Parametric Mapping (SPM8, Institute of Neurology, UK) and Matlab (Mathworks, USA). First, we reoriented the images manually to set their origins to the anterior commissure. Then the T1 image of every subject was segmented into gray matter (GM), white matter (WM) and cerebrospinal fluid (CSF)^31^. Meanwhile, the total volume of GM, WM, and CSF were estimated and reported by VBM8. After that, a high-dimensional non-linear DARTEL (Diffeomorphic Anatomical Registration Through Exponentiated Lie^32^) algebra was performed for normalization and modulation of the GM and WM. The modulated GM maps were smoothed with an 8 mm full-width at half maximum Gaussian kernel.

VBM statistics were conducted in SPM8 using a 2 × 2 full factorial model defining diagnosis (HC; MDD) and genotype (low risk; high risk) as two factors, while using age, sex, years of education and total intracranial volume as covariates to remove their effects on variance. An explicit grey matter mask was used to prevent effects occurring outside grey matter. We assessed the main effect of diagnosis, the main effect of genotype and the interaction effect, the disease effects within low-risk or high-risk subgroup using F contrasts. All the significant results at *p* < 0.001 (uncorrected) were reported. With DPABI^33^ software, AlphaSim program based on Monte Carlo simulation was applied for multiple comparison corrections^34^, and the current version has solved the bug raised by Eklund et al^35^ with the real smoothness in the three directions calculated from the residuals. 5000 simulations for every statistic map were conducted and strict threshold was applied (voxel-wise *p* < 0.001 and cluster-wise *p* < 0.05, two-tailed). Anatomical regions emerging from VBM analysis were labeled according to Anatomical Automatic Labeling (AAL) atlas and visualized with BrainNet Viewer^36^. Additionally, we used xjView toolbox (http://www.alivelearn.net/xjview) together with Easy Volume toolbox (http://www.sbirc.ed.ac.uk/LCL/LCL_M1.html) to extract mean volumes of the clusters that had shown significant effects in VBM analysis.

### Graph theory analysis

We primarily applied Graph Analysis Toolbox (GAT^37^) to implement graph analysis of the structural networks of MDD patients and HCs in the high-risk subgroup. First, 90 regions of interest (ROIs) based on the cerebral part of Anatomical Automatic Labeling (AAL) atlas were defined, masks of them were generated using WFU PickAtlas Toolbox^38^ and were resliced to the same dimension as modulated, normalized GM images from VBM preprocessing step. REX code (http://web.mit.edu/swg/software.htm) was used to extract the mean volume of every ROI. Second, the extracted volume data of 90 ROIs were used to construct structural correlation networks. A 90 × 90 correlation matrix (R) was generated in each group with each entry r_ij_ representing the Pearson correlation coefficient between the volume of ROI *i* and *j*. Then each R matrix was converted to a binary association matrix (A), with entry a_ij_ considered 1 If *r*_*ij*_ bigger than a specific threshold and zero otherwise. Thresholding and construction of structural networks were carried out at a range of network densities (D_min_: 0.24: 0.02: 0.5), across which also group comparison was conducted. The lower limit of the range was the calculated minimum density allowing all nodes to be fully connected with each other, and the upper limit of the range was set to 0.5, as densities above 50% are not sparse anymore and thus not biologically meaningful for brain networks. Accordingly, a graph (G) including 90 nodes was derived from A, and regions i and j were regarded as connected if g_ij_ is unity. Network degree (E) was equal to the number of edges, and the network density (D) was used to present the fraction of actually existent edges to all possible links. Third, topology of the global network, such as small-worldness, normalized clustering and normalized path length, were measured using codes in the Brain Connectivity Toolbox^39^ at both the network and regional level. The small-worldness of a network has two key measurements, clustering coefficient (C) and characteristic path length (L). The clustering coefficient is a measure of network segregation and is represented as the mean value of clustering coefficients, while the path length is a measure of network integration and is defined as the mean shortest path length between all pairs of nodes in the network. All the measurements were compared to the consistent mean values of a benchmark random graph so as to appraise the topology of the brain network, and m (the number) null networks were generated for normalization of C and L. Normalized C and normalized L were calculated as C/C_rand_ and L/L_rand_, and small-wordness was obtained as [C/C_rand_]/[L/L_rand_] (C_rand_ and L_rand_ were the mean clustering coefficient and the characteristic path length of the m random networks.). Compared to random network, small-world network is characterized by significantly higher clustering coefficient (C/C_rand_ ratio greater than 1) as well as similar characteristic path length (L/L_rand_ ratio close to 1). In terms of regional network characteristics, nodal clustering, degree, and betweenness were estimated and compared at the minimal density for significant brain regions from VBM analyses. Nodal clustering quantifies how close its neighbors are to being a clique, nodal degree is defined as the number of the edges from a node to others, and nodal betweenness is represented as the proportion of all shortest paths in the network that pass through a given node. The regional measures were normalized by the mean network value respectively, and then compared between groups. Forth, the within-group difference of network topology was identified, the between-group difference of global and regional network measures were tested using non-parametric permutation test with 1000 repetitions.

The VBM and GAT analyses were repeated more than twice with the same results.

### Statistical analysis

Statistical analyses of clinical data were conducted with SPSS19.0 (SPSS Inc., Chicago, IL, USA). Normal distribution test was performed using One-Sample Kolmogorov–Smirnov Test in groups of MDD and HC separately, and Levene’s Tests were used to test the homogeneity of variances. (Table S1 in the Supplementary Materials.) The differences between the MDD and HC were evaluated using Mann-Whitney Test (age and years of education, which did not coincide with normal distribution), two-sample *t*-Test (total brain volume) and Chi-square test (gender and genotype).

## Results

### Demographics

No significant difference of age, gender, years of education, total brain volume or distribution of SNP genotypes was found between groups of MDD and HC (Table 1).

**Table 1:** Demographics of MDD patients and HCs

		MDD	HC	*χ²/Mann–Whitney U/t*	p value
Male		60 (41.7%)	53 (47.7%)	0.939	0.332
Age		28.17 ± 5.91	27.63 ± 5.43	7548.000	0.446
Years of education		15.29 ± 2.55	15.51 ± 2.95	7233.500	0.188
Total brain volume (ml)		1410.42 ± 132.17	1429.71 ± 126.45	−1.177	0.240
rs1799724	CC	110 (76.4%)	83 (74.8%)	0.089	0.766
	CT/TT	34 (23.6%)	28 (25.2%)		

### VBM analysis

**Fig. 1:**
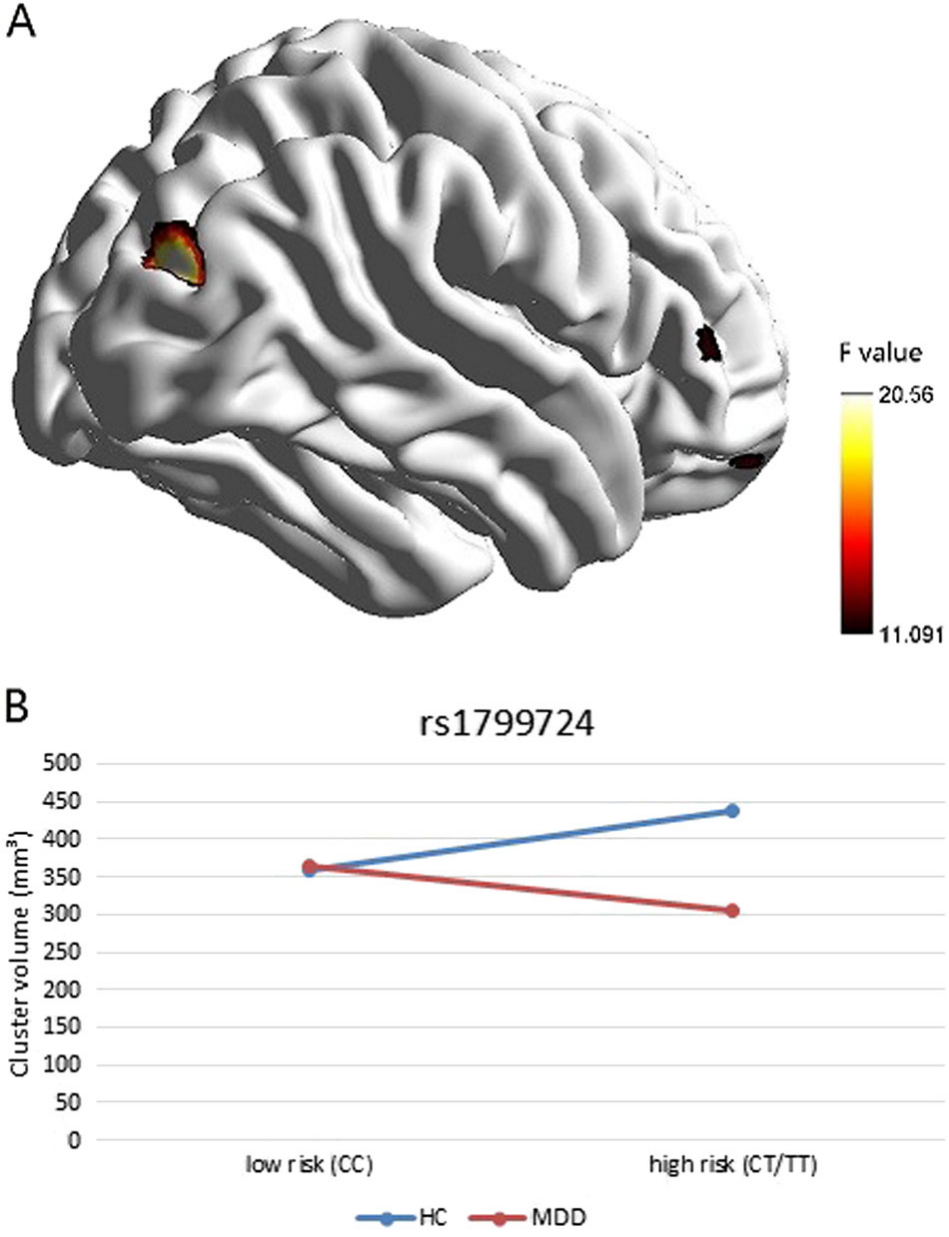
The interaction effect of diagnosis x genotype at rs1799724 was located in right superior occipital gyrus in VBM analysis (**a**) clusters in the brain (*p* < 0.001); **b** line chart showing interaction effect (*p* < 0.05, AlphaSim corrected)

The interactive effects of diagnosis and rs1799724 primarily influenced right superior occipital gyrus, the orbital part of right middle frontal gyrus and the triangular part of the right inferior frontal gyrus.(Table 2) After AlphaSim correction, however, diagnosis x genotype interaction was exclusively localized to the visual cortex (right superior occipital gyrus, Fig. 1), and the same region also showed reduced volume in patients with MDD than HC (main effect of diagnosis, Table 2, Fig S1 in the Supplementary Materials). The effect of rs1799724 mainly located in brain regions of left angular gyrus, left middle occipital gyrus, the medial part of left superior frontal gyrus, right supplementary motor area and left precuneus. (Table 2) Among these regions, left angular gyrus was focalized after correction for multiple comparisons, with high-risk individuals showing reduced volume (Fig S2 in the Supplementary Materials). From post-hoc T-Test, low-risk MDD revealed decreased volume in left inferior parietal gyrus (*p*_AlphaSim_ < 0.05), left angular gyrus, right precuneus gyrus, left inferior parietal gyrus and left supplementary motor area than low-risk HC (Table 2, Fig S3 in the Supplementary Materials), while depressive individuals showed less volume in right superior occipital gyrus (*p*_AlphaSim_ < 0.05) and the orbital part of right middle frontal gyrus than HC in the high-risk subgroup (Table 2, Fig S4 in the Supplementary Materials).

**Table 2:** Significant brain regions in VBM analysis (*p* < 0.001, uncorrected)

Anatomical region	Cluster size	*F* value	Low-risk HC (mm³)	High-risk HC (mm³)	Low-risk MDD (mm³)	High-risk MDD (mm³)	MNI coordinates
Interaction of diagnosis and rs1799724							
Right superior occipital gyrus^a^	174	20.57	357.9 ± 125.9	437.3 ± 116.1	364.4 ± 113.8	304.6 ± 101.9	22.5, −82.5, 22.5
Right middle frontal gyrus, orbital part	14	13.55	28.9 ± 8.7	32.5 ± 9	31.1 ± 10	23.2 ± 10.1	24, 57, −18
Right inferior frontal gyrus, triangular part	15	11.79	23.5 ± 6.1	18.8 ± 5.4	20.9 ± 5.3	22.3 ± 6.4	54, 33, 7.5
Main effect of diagnosis							
Right superior occipital gyrus^a^	124	19.91	261.4 ± 100.5	323.2 ± 92	262.1 ± 90.4	216 ± 83.7	19.5, −85.5, 21
Main effect of rs1799724							
Left angular gyrus^a^	526	20.07	528.8 ± 107.6	451.9 ± 88.4	488 ± 96	438.7 ± 116.4	−40.5, −66, 57
Left middle occipital gyrus	23	15.23	32.6 ± 15.6	44.1 ± 17.5	34.7 ± 15.7	44.1 ± 17.9	−13.5, -88.5, −6
Left middle occipital gyrus	17	12.16	0.2 ± 0.3	0.1 ± 0.1	0.2 ± 0.2	0.1 ± 0.2	−33, -97.5, 19.5
Left superior frontal gyrus, medial part	62	12.10	77 ± 15.8	67.4 ± 18.5	72.4 ± 18	65.4 ± 18.2	−1.5, 28.5, 43.5
Right supplementary motor area	20	12.40	26.1 ± 8.1	19.8 ± 9.6	22.4 ± 9.2	21.1 ± 9.2	3, -19.5, 51
Left precuneus	49	13.53	17.3 ± 13.1	12 ± 9.3	15.7 ± 11.5	13.6 ± 11.7	−3, −46.5, 66
Disease effects in low-risk subgroup							
Left inferior parietal gyrus^a^	286	17.37	440.1 ± 80.3	405.1 ± 84.7	398.2 ± 73.7	383 ± 75.3	−42, −51, 51
Left angular gyrus	84	14.45	204.1 ± 51	178.1 ± 58	180.9 ± 50.1	183 ± 57.6	−46.5, −55.5, 33
Right precuneus gyrus	83	13.85	187.2 ± 45.4	165.6 ± 38.9	159.4 ± 44.2	171.7 ± 40.3	12, −58.5, 43.5
Left inferior parietal gyrus	62	14.26	57.5 ± 14.2	51.3 ± 9.1	50.8 ± 12	53.3 ± 11.5	−58.5, −45, 51
Left supplementary motor area	14	12.32	6.8 ± 5.4	5.2 ± 4.4	5.2 ± 5.3	4.7 ± 4.1	1.5, 10.5, 45
Disease effects in high-risk subgroup							
Right superior occipital gyrus^a^	227	25.39	449.8 ± 145	543 ± 142.8	455.1 ± 132.2	384.6 ± 115.8	21, −84, 22.5
Right middle frontal gyrus, orbital part	24	14.14	49.9 ± 12.8	54.3 ± 12.5	51.8 ± 14.8	40.6 ± 16.8	25.5, 55.5, −18

^a^Parts of these clusters are also significant at *p* < 0.05, AlphaSim corrected

### GAT analysis in the high-risk subgroup

**Fig. 2:**
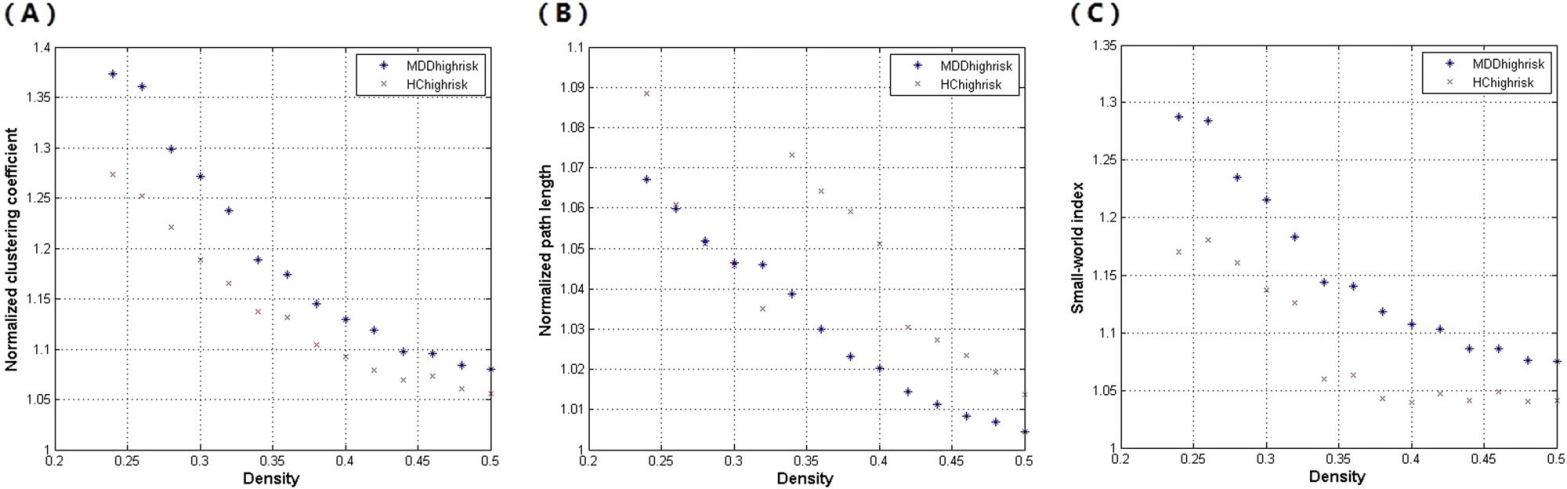
The global network is measured at different network density. Normalized clustering (**a**), normalized path length (**b**), and small-world index (**c**) of the major depressive disorder (MDD) and healthy control (HC) networks in the high-risk subgroup

**Fig. 3:**
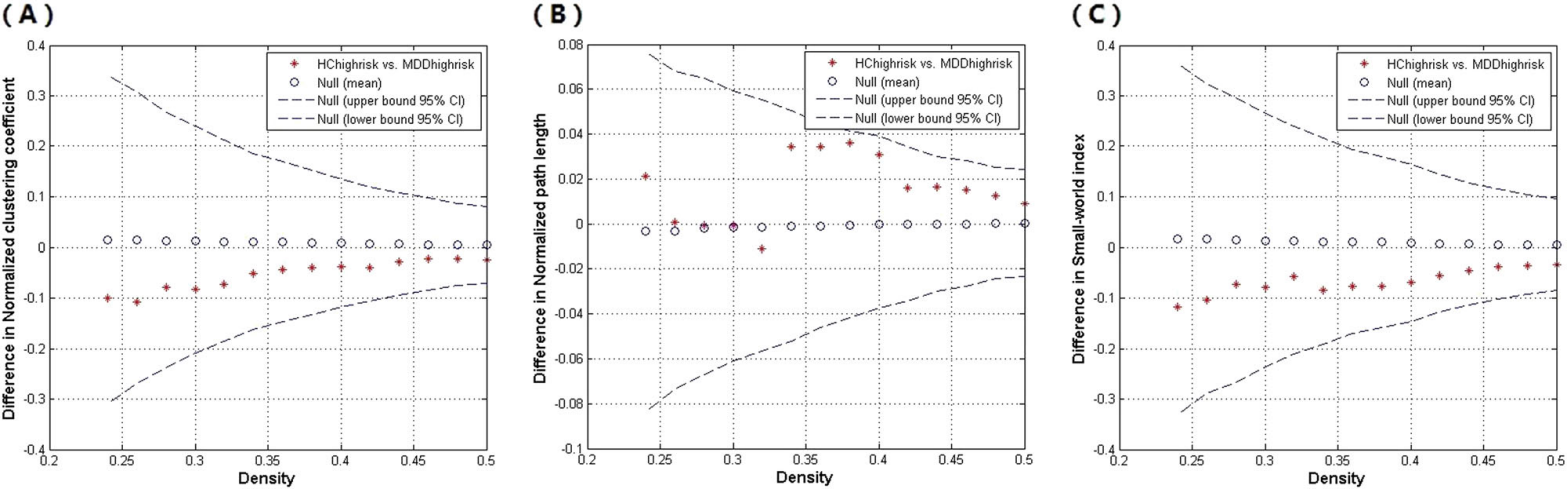
The global network differences between major depressive disorder (MDD) and healthy control (HC) participants in the high-risk subgroup at different network density. The 95% confidence intervals (CI) and group differences in normalized clustering (**a**), normalized path length (**b**), and small-world index (**c**). The * marker shows the difference between the high-risk subgroup of MDD and HC; the * signs falling outside of the confidence intervals indicate the densities in which the difference is significant at *p* < 0.05. The positive values show HC_highrisk_ > MDD_highrisk_ and negative values show HC_highrisk_ < MDD_highrisk_

The global network topology of shortest path length, clustering coefficient and small-worldness were measured across a range of densities (Dmin: 0.24: 0.02: 0.5) and compared within and between groups. In each group of high-risk MDD and HC, characteristic path length approached to 1, while clustering coefficient and small-worldness were higher than 1, indicating a small-world organization (Fig. 2). However, no significant difference of clustering coefficient, shortest path length or small-worldness was found between the two groups of high-risk MDD and HC, as all the difference values appeared within the confidence interval in Fig. 3. Regional network measures of right superior occipital gyrus, left angular gyrus and left inferior parietal gyrus were compared at the minimal density (0.24), nevertheless, no significant difference of nodal clustering, degree or betweenness was found between groups (Table S1 in the Supplementary Materials).

## Discussion

In the present study, we report three major findings. Firstly, TNF polymorphism (rs1799724) has a predominant effect on the structure of the visual cortex in the presence of depression. Secondly, as there is no notable effect on the structural covariance networks, it is likely that the morphological aberration is late in onset and highly localized in nature. Thirdly, in medication-free MDD patients, grey matter changes are of small effect size and limited in distribution^40^.

Our findings demonstrate that a polymorphism, which is potentially linked to higher transcription of TNF-α, is associated with structural changes in the occipital cortex, especially in the presence of depression. These results add to the burgeoning literature implicating visual stimulus processing deficits^41^ and occipital cortex abnormalities in MDD^42^. While gray matter reduction in occipital cortex has been reported inconsistently in MDD^43–45^, prior reports highlight reduced functional connectivity^46–51^, perfusion deficits^52,53^ and abnormal structure^54–56^ of occipital cortex in MDD. Given that our sample comprised largely of drug-naïve first episode MDD, and the fact that only high-risk group had the MDD-related reduction in occipital cortex volume, this association is unlikely to be explained by the use of antidepressants or duration of illness.

The T allele of rs1799724 has the higher transcriptional activity for TNF^17^. Higher levels of TNF-α^57^ relates to depression-like symptoms^58^, thus indicating that the carriers of T form a high-risk group. Of interest to our findings, TNF-α has been showed to play an important role in the synaptic plasticity to contribute to the anatomy of visual cortex. In particular, in the absence of TNF-α, compensatory adjustment to monocular deprivation does not occur in the non-deprived eye^59^. TNF-α has been shown to mediate experience-dependent plasticity through homeostatic synaptic scaling^24^ as well as long-term potentiation^59^. Of further relevance to depression where diurnal variation in emotion, cognition, and motivation occurs in a prominent fashion, the notable diurnal variation has been reported in the expression of TNF-α receptors in the murine visual cortex, as a response to daylight^60^. As the visual system is used differently in day and night, the levels of some cytokines, including TNF-α, and the number of their receptor cells varies in different time of day^60^. TNF-α expression and receptor cells increased in the dark compared to the light. The alteration in visual cortex and related emotion and cognition of MDD patients may be explained by varying TNF-α and other cytokines partly. Taken together, our observation of MDD × genotype interaction indicates that in a subgroup of depressed subjects with the high-risk variant, the trophic effects of TNF-α on visual cortex may be disrupted.

We studied structural covariance using graph theory, to detect the effect of TNF polymorphism on the coordinated maturation of distributed, large-scale morphometric networks. We observed no MDD × genotype interaction on the structural covariance across the entire brain. Morphometric networks with tightly co-varying patterns of brain anatomy are likely to have mutually shared trophic influences such as functional co-activation or expression of plasticity modulating factors^61^. The lack of disruption in structural covariance in depressed subjects with the high-risk genotype suggests that the effect of TNF-α may be either temporally restricted (e.g., periods of inflammatory activity or critical window of development) and/or spatially restricted to the occipital cortex. It is important to consider this in light of our observation that there was a small, statistically insignificant effect of diagnosis x genotype interaction in other brain regions in our sample. Such spatially limited grey matter deficits are in line with several VBM studies investigating a comparable sample (first episode, unmedicated subjects with MDD)^40^.

There are several limitations that need to be considered when interpreting the results of this study. Firstly, as a transcriptional regulator, rs1799724 is not the only SNPs in the promoter region of TNF-α; nevertheless, studies have suggested a link between rs1799724 and neurodegenerative/neurovascular disorders such as Alzheimer’s disease^18,19^, stroke^20^ and depression after stroke^21^. We plan to undertake further neuroimaging studies with other SNPs of TNF-α, subsequent to appropriate approvals to study their individual effects as well as interactions among the various polymorphic variants. Secondly, we lacked functional data on task performance (especially for visual stimulation) and neuropsychological data relevant to visual processing, to further study the implications of the observed structural change. This is one of our goals for the future studies. Thirdly, it is possible that there are other potential confounding factors mediating the observed relationship between TNF polymorphism and brain structure in depression. We lacked sufficient quantitative data on smoking, alcohol and drug use, stress and childhood trauma, that may also influence the brain structure.

In conclusion, the TNF-α SNP, rs1799724 (TNF-850 or 857 C/T), has a notable effect on the brain structure in both patients with MDD and healthy controls, with disease-related effects localizable to the occipital cortex. Our observations raise questions regarding the disruptions in the trophic role played by pro-inflammatory cytokine system in depression. Our results are highly relevant to understanding the brain-basis of anti-inflammatory treatments in depression, as well as isolating sub-groups of depressed subjects who may differ in cortical anatomy, genotype as well as response to novel therapeutics.

## Electronic supplementary material

Supplementary Materials0703
