# Supplementary material for: Effects of tumor necrosis factor-α polymorphism on the brain structural changes of the patients with major depressive disorder
Source: Transl Psychiatry. 2018 Oct 11;8:217. doi: 10.1038/s41398-018-0256-x (PMC6181976; doi:10.1038/s41398-018-0256-x)
Supplement: Supplementary file 1 — Supplementary Materials0703 [file 41398_2018_256_MOESM1_ESM.docx]

**Fig S1 The main effect of diagnosis in VBM analysis (p<0.001).**

**Fig S2 The main effect of genotype in VBM analysis (p<0.001).**

**Fig S3 Disease effects of low-risk subgroup in VBM analysis (p<0.001).**

**Fig S4 Disease effects of high-risk subgroup in VBM analysis (p<0.001).**

**Table S1 Tests of Normal Distribution and Equality of Variances**

**Table S2 Regional network differences between major depressive disorder (MDD) and healthy control (HC) participants in the high-risk subgroup at the minimal density (p value).**

**
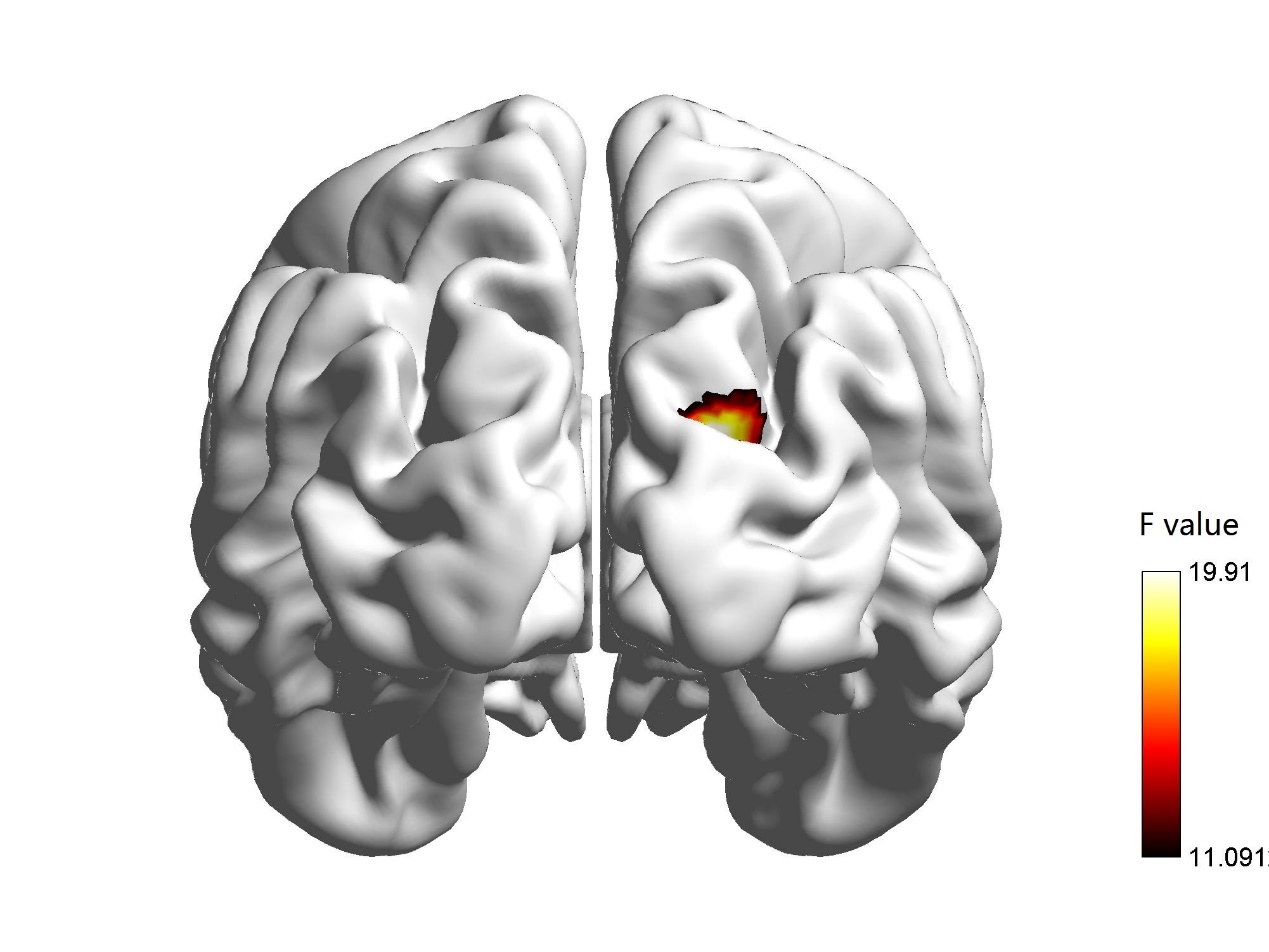
**

**Fig S1 The main effect of diagnosis in VBM analysis (p<0.001).**

**
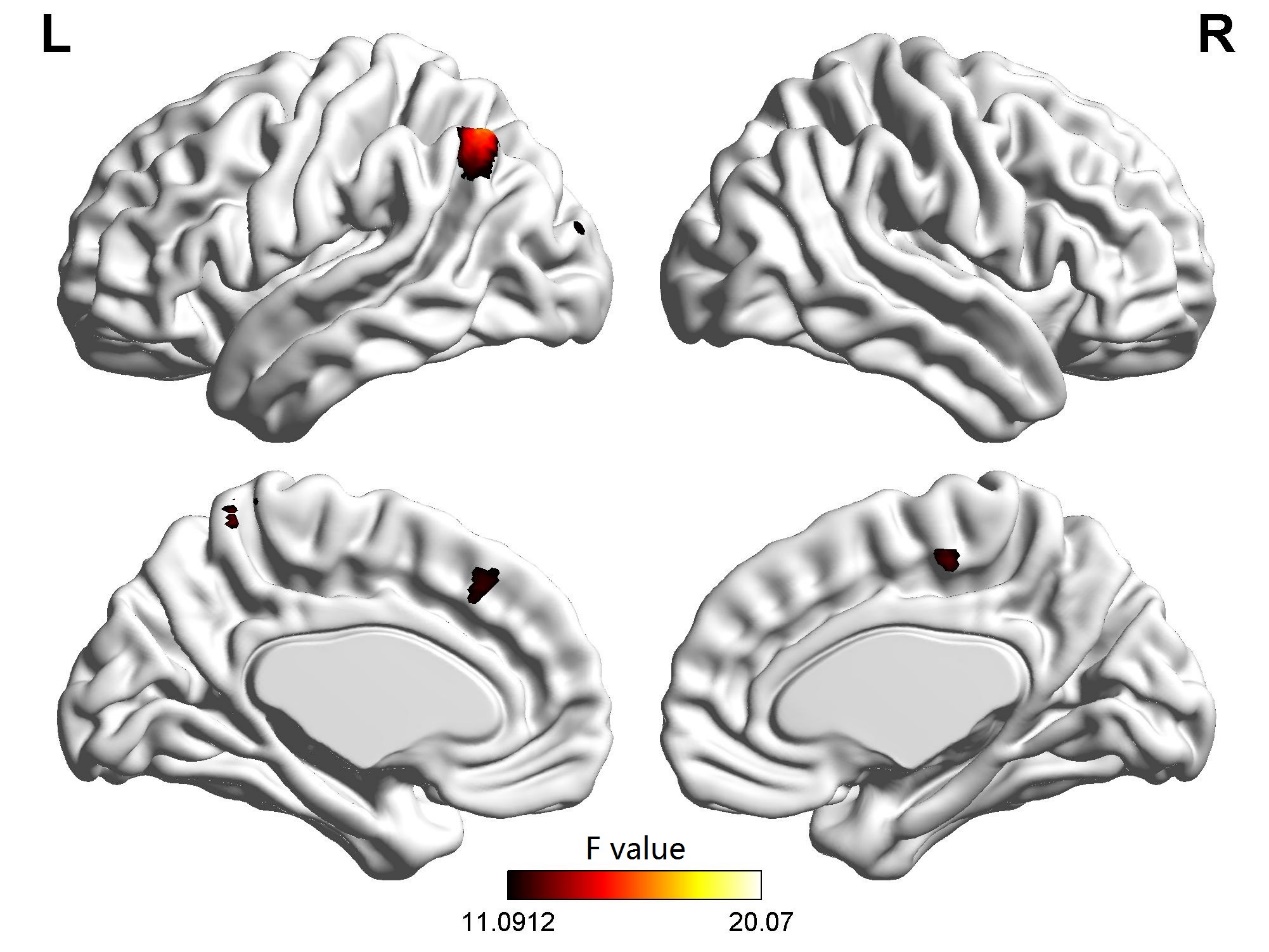
**

**Fig S2 The main effect of genotype in VBM analysis (p<0.001).**

**
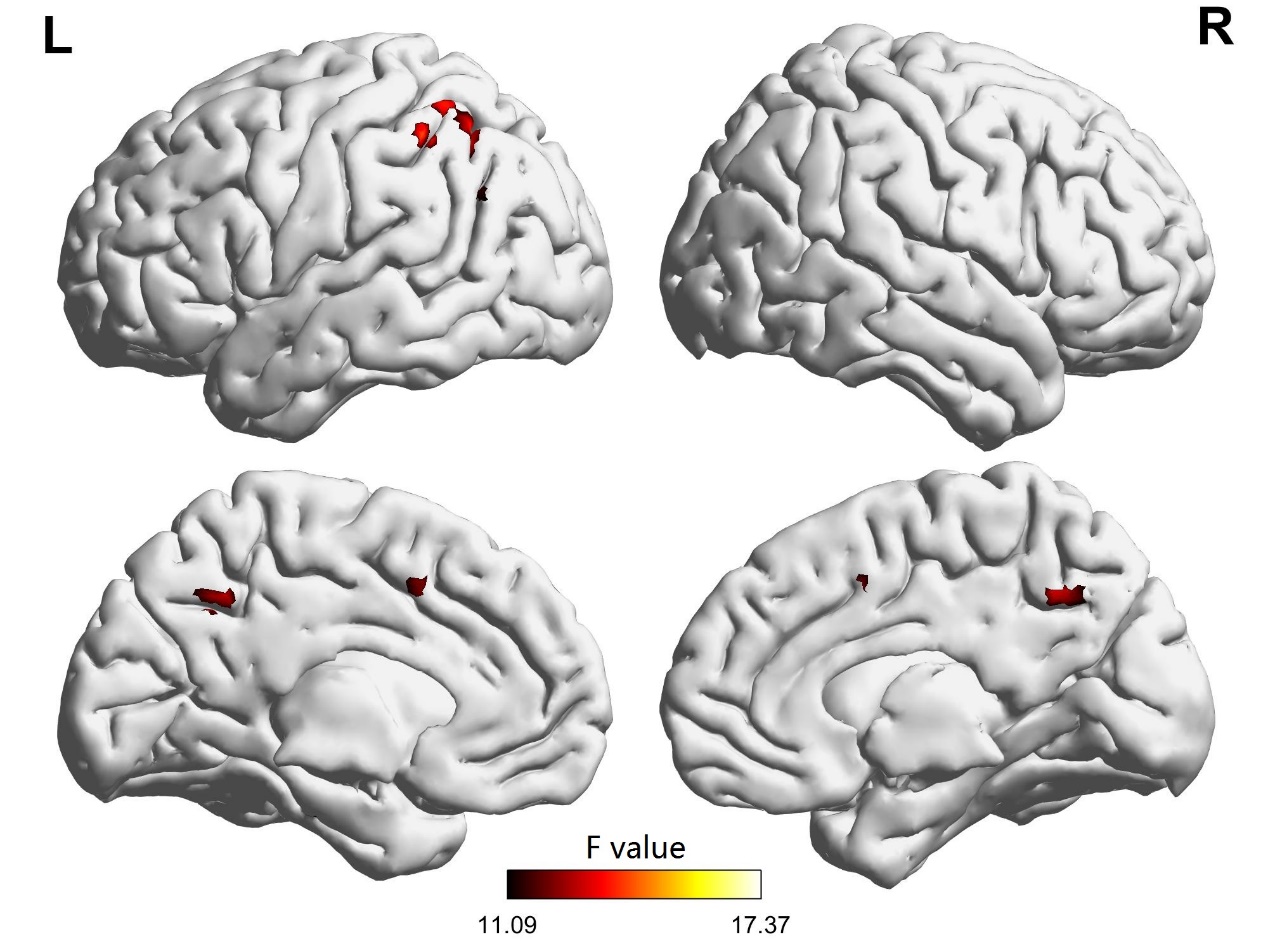
**

**Fig S3 Disease effects of low-risk subgroup in VBM analysis (p<0.001).**

**
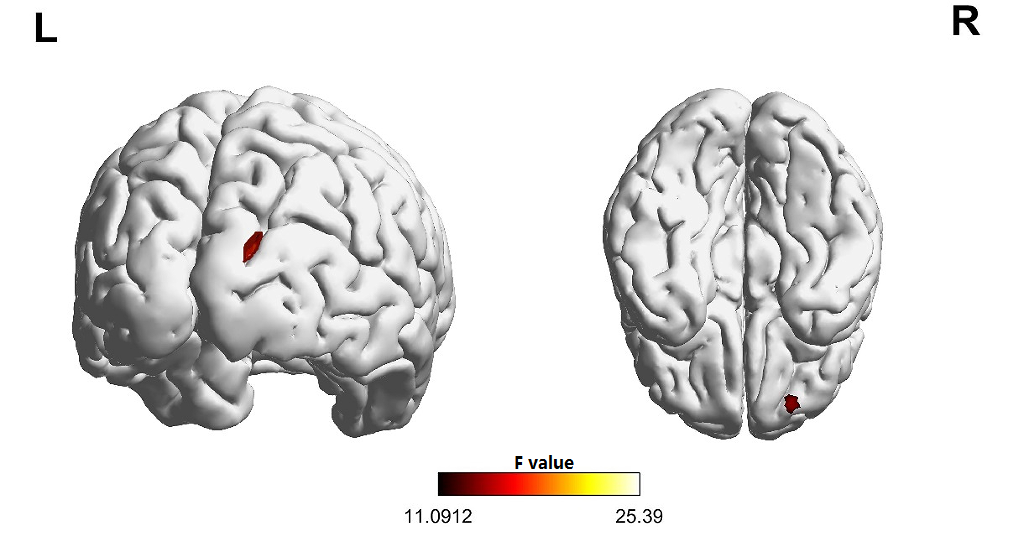
**

**Fig S4 Disease effects of high-risk subgroup in VBM analysis (p<0.001).**

| \| **Table S1 Tests of Normal Distribution and Equality of Variances** \| \| \| \| \| \| --- \| --- \| --- \| --- \| --- \| \|  \| **Kolmogorov-Smirnov Test (P value)** \| \| **Levene's Test** \| \| \| **MDD** \| **HC** \| **F value** \| **P value** \| \| Age \| 0.057 \| 0.000 \| 1.140 \| 0.287 \| \| Years of education \| 0.000 \| 0.000 \| 2.863 \| 0.092 \| \| Total brain volume (ml) \| 0.200 \| 0.090 \| 0.443 \| 0.506 \|   **Table S2 Regional network differences between major depressive disorder (MDD) and healthy control (HC) participants in the high-risk subgroup at the minimal density (P value)** | | | |
| --- | --- | --- | --- | --- | --- | --- | --- | --- | --- | --- | --- | --- | --- | --- | --- | --- | --- | --- | --- | --- | --- | --- | --- | --- | --- | --- | --- | --- | --- | --- | --- | --- |
|  | **Normalized clustering** | **Normalized**  **degree** | **Normalized betweenness** |
| Right superior occipital gyrus | 0.503 | 0.519 | 0.561 |
| Left angular gyrus | 0.706 | 0.15 | 0.251 |
| Left inferior parietal gyrus | 0.947 | 0.093 | 0.378 |
